# Case with a Nonreassuring Fetal Status Induced by Massive Hematemesis due to Mallory-Weiss Tear That Required Emergency Cesarean Section at 38 Weeks' Gestation

**DOI:** 10.1155/2015/762463

**Published:** 2015-12-31

**Authors:** Takashi Suzuki, Maiko Wagata, Hiroko Konno, Takahiro Ito, Yuichi Torii, Takeshi Murakoshi

**Affiliations:** Division of Perinatology, Fetal Diagnosis and Therapy, Maternal and Perinatal Care Center, Seirei Hamamatsu General Hospital, 2-12-12 Sumiyoshi, Hamamatsu 430-8558, Japan

## Abstract

We describe a rare case of Mallory-Weiss tear with massive hematemesis at 38 weeks' gestation. A 35-year-old woman presented with epigastralgia followed by massive hematemesis. An emergency endoscopy indicated active pulsatile bleeding at the esophagocardial junction. Although an emergency endoscopic hemostasis was successful, late decelerations without acceleration on cardiotocogram were observed. Therefore, the patient underwent emergency cesarean section, along with blood transfusion, following the endoscopic hemostasis. The hemoglobin level just before the operation was 5.1 g/dL. We suspected that massive hematemesis induced maternal acute anemia and hypovolemia, which resulted in a nonreassuring fetal status. Hence, urgent endoscopic hemostasis, adequate blood transfusion, and emergency cesarean section were needed. Mallory-Weiss tear during the third trimester may have a possibility of massive hematemesis and urgent blood transfusion, emergency endoscopic hemostasis, and emergency cesarean section may be needed.

## 1. Introduction

Mallory-Weiss tear (MWT), or Mallory-Weiss syndrome, involves a triad of vomiting, hematemesis, and alcoholism and was first reported by Mallory and Weiss in 1929 [1]. It is characterized by the presence of longitudinal mucosal lacerations in the distal esophagus and proximal stomach, which are usually associated with forceful retching. During pregnancy, MWT may develop due to hyperemesis gravidarum in the first trimester, and the volume of bleeding is such that it can be controlled through spontaneous hemostasis. However, reports of third trimester of pregnancy with a complication of MWT are scarce, and they are complicated with scleroderma [2, 3].

In the present report, we describe a very rare case of MWT with massive hematemesis at 38 weeks' gestation without complication of scleroderma. Although the patient was successfully treated with emergency endoscopic hemostasis, an emergency cesarean section and blood transfusion were nevertheless required due to the observation of a nonreassuring fetal status (NRFS) on cardiotocography. Following this treatment, both the mother and newborn were discharged in a good condition without any complications.

Our case and previously reported cases suggest that MWT during third trimester of pregnancy may cause massive hematemesis which is different from the one during the first trimester and we should keep it in mind and prepare for it.

## 2. Case Presentation

A 35-year-old Japanese woman (body weight: 59.3 kg; gravida 2, para 1 with normal vaginal delivery) presented to our outpatient department with epigastralgia and nausea. Her medical history indicated that she was previously followed up for suspected multiple sclerosis, restless leg syndrome, and Hashimoto's thyroiditis, all of which were well controlled. There was no history of scleroderma. During perinatal visits, she exhibited iron deficiency anemia and was prescribed tablets of ferrous fumarate. Her hemoglobin level on the day before the current admission was 8.9 g/dL.

During her perinatal visit, massive hematemesis was noted on physical examination, which was suspected to be due to bleeding from the upper gastrointestinal tract, and the patient was hence urgently admitted. At that time, her blood pressure was 106/60 mmHg, and her heart rate was 111 beats/min. She admitted emergently in the maternal-fetal intensive care unit, and laboratory examination was conducted, which was shown in Table 1. No major abnormal data including coagulation profiles were observed.

**Figure 1:**
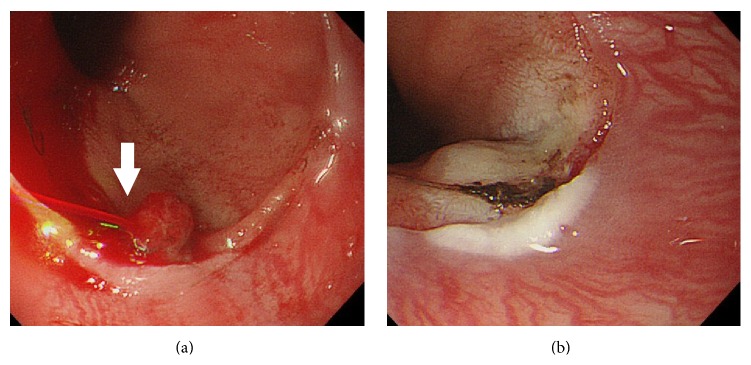
Endoscopic findings before and after hemostasis. (a) Active pulsatile bleeding from the esophagocardial junction (arrow). (b) Hemostasis by cauterization along with spraying of 10,000 IU of thrombin.

**Figure 2:**
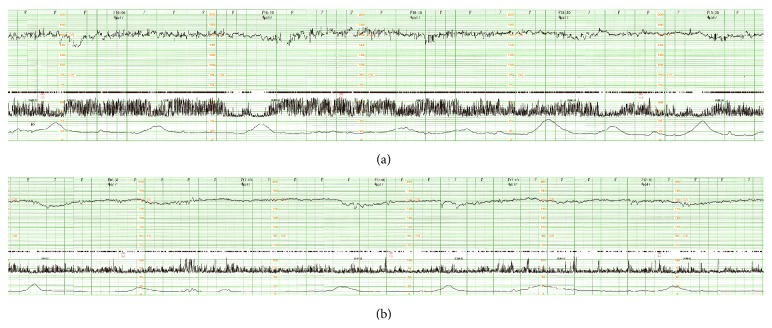
Cardiotocogram indicates a nonreassuring fetal status. (a) Cardiotocogram on admission. The fetal heart rate is 160 beats/min, with minimal variability and recurrent mild to severe late decelerations without any acceleration. (b) Cardiotocogram after endoscopic hemostasis. The findings are similar to those observed in (a). However, late decelerations are also noted during every mild uterine contraction, which is not observed on admission.

**Figure 3:**
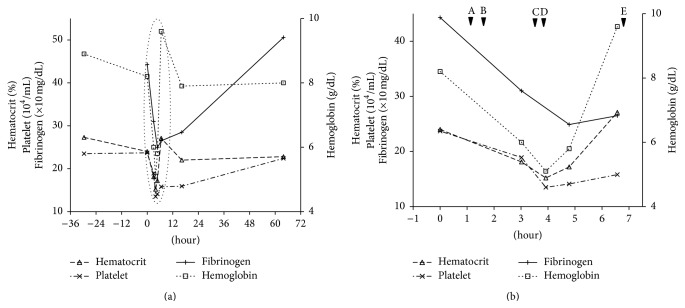
Chronological changes in laboratory data and a review of the procedures during the perioperative period. “0” on the horizontal axis indicates the time of admission. The right vertical axis represents the hemoglobin level, whereas the left vertical axes represent the hematocrit, platelet, and fibrinogen levels. (a) Overall view from the day before admission to the third postoperative day. The dotted circle area indicates the perioperative period. (b) Magnification of the dotted circle area from Panel (a). The upper-case alphabet with an arrow head indicates the time of the procedure. (A) Time of endoscopic hemostasis. (B) Observation of nonreassuring fetal status on cardiotocography. (C) Time of commencing cesarean section. (D) Time of commencing red blood cell transfusion. (E) Time of commencing fresh frozen plasma transfusion.

Following rapid intravenous infusion with 2000 mL of colloids and crystalloids, emergency endoscopy was performed, which indicated pulsatile bleeding from the esophagocardial junction and suggested a diagnosis of MWT. Hence, hemostasis was successfully performed through endoscopic cauterization and spraying of 10,000 IU of thrombin (Figure 1). The total amount of blood loss before hemostasis was unknown. Nevertheless, although the hemostasis was successful, the fetal heart rate remained at 160 beats/min. Moreover, minimal variability along with mild to severe late decelerations without any acceleration was noted on the cardiotocogram (Figure 2), which was similar to that observed on admission, thus indicating NRFS that prompted an emergency cesarean section. As her hemoglobin level was 5.1 g/dL just before the operation, red blood cell (RBC) infusion was initiated during the surgery. The newborn was a female, with a body weight of 2742 g and Apgar scores of 8 and 9 at 1 and 5 minutes, respectively. The pH of the umbilical artery was 7.388, pO_2_ was 14.9, pCO_2_ was 40.8, BE was −0.9, and hemoglobin level was 12.6 g/dL. As the maternal fibrinogen and hemoglobin levels during the operation were 249 mg/dL and 5.8 g/dL, respectively, transfusion of fresh frozen plasma (FFP) was initiated.  Figure 3 showed a chronological review of the laboratory data and procedures. In total, 6 units each of RBCs and FFP were transfused. Following treatment, both the mother and newborn were discharged in a good condition without any complications. After the nine months of follow-up, the infant was alive and well without any developmental delay.

## 3. Discussion

Cases of massive hematemesis due to MWT in the third trimester are rare. To our knowledge, during the peripartum period, only two cases of massive hematemesis during the third trimester with blood transfusion [2, 3] and another one case during the postpartum [4] have been reported in the literature. During pregnancy, MWT may usually develop in the first trimester due to hyperemesis gravidarum. Nevertheless, even in Williams Obstetrics [5], no information has been provided regarding the frequency of MWT during pregnancy.

The cause of the MWT remains unknown in the present case. The contributing factors among such cases generally include vomiting, straining when passing stools or while lifting, coughing, hiatal hernia which is frequent in pregnant women, epileptic convulsions, hiccups under anesthesia, closed-chest massage, blunt abdominal injury, and gastroscopy [6–9]. However, the current case did not have any history of alcohol consumption, chronic nausea, and vomiting, constipation, or convulsions before hematemesis. There was no abnormal laboratory data on coagulation profile shown in Table 1. There was a report that medical history of scleroderma is a contributing factor [2, 3]; however, there is no known medical history of scleroderma or any other pathophysiological histories in this case. Hiatal hernia was not recognized during the endoscopy. Furthermore, tears occurring in the esophagus or overlying the esophagocardial junction, like our case, are more often associated with the absence of demonstrable hiatal hernia, which implies that hiatal hernia is less likely [8]. Spontaneous rupture of the esophagus, or Boerhaave's syndrome, associated with high morbidity and mortality, is fatal in the absence of therapy and is a spontaneous perforation of the esophagus that results from a sudden increase in intraesophageal pressure combined with negative intrathoracic pressure (e.g., severe straining or forceful vomiting). This syndrome is the proximity with MWT [8, 10, 11]; however, this case was not esophageal rupture but laceration on the esophagocardial junction.

Normocardia along with minimal variability and recurrent late deceleration without any acceleration was observed on cardiotocography in the present case. Animal studies have indicated that late decelerations is the first sign of fetal deterioration, which may occur with a slight but significant decrease in fetal PaO_2_ without any changes in pH, even though accelerations in fetal heart rate can still be observed. The loss of accelerations in the fetal heart rate occurs at a later stage and is associated with a significant reduction in fetal pH and PaO_2_ [12]. However, this case did not show reduction of fetal pH and PaO_2_.

In this case, we believe that the NRFS may have been caused by acute massive hematemesis followed by acute severe anemia with hypoxemia, even though the fetal pH was not significantly reduced. Hypovolemia may be another possibility of NRFS; however, there was no improvement in fetal heart rate following the rapid intravenous infusion of colloids and crystalloids in this case. Hence, urgent endoscopic hemostasis, adequate blood transfusion, and emergency cesarean section were required.

The estimated blood loss prior to endoscopic hemostasis was approximately 1.5–1.8 L. This was primarily derived based on the shock index of almost one (systolic blood pressure: 106, pulse rate: 111 beats/min) at the time of admission, which suggests that the blood loss was approximately 1.5 L. In addition, the change in the hemoglobin level from 8.9 to 5.1 g/dL suggests that the estimated blood loss was approximately 1.8 L; moreover, based on this change, the overall loss of hemoglobin was estimated as 157.7 g. Two units of RBC prepared by the Japanese Red Cross contain 53.2 g of hemoglobin in 280 mL. Hence, we considered that the transfusion of 6 units of RBCs would be appropriate. As the fibrinogen level was 249 mg/dL during the operation and was also relatively low during the intrapartum period and just after endoscopic hemostasis, we decided that the amount of FFP to be transfused should be similar to that of RBCs; hence, 6 units each of RBCs and FFP were transfused in total. Two case reports of MWT during the third trimester also conducted blood transfusion [2, 3].

In cases of preterm pregnancy with massive hematemesis, particularly among those wherein the continuation of pregnancy is preferable, there may be sufficient time to assess the responsiveness to endoscopic hemostasis and blood transfusion (especially whether the NRFS can be changed to a reassuring fetal status), and the pregnancy can be continued accordingly, which is different from massive bleeding of placenta previa or placental abruption because the way of hemostasis for these two diseases is usually the termination of pregnancy with cesarean section.

Considering our case and reported cases [2, 3] together, MWT during the third trimester may have a possibility for massive hematemesis, and we should consider to prepare for urgent blood transfusion, emergency endoscopic hemostasis, and cesarean section, even though MWT in pregnancy is very uncommon.

## Figures and Tables

**Table 1:** Laboratory data on admission.

White-cell count (/mL)	17690
Erythrocyte count (10^4^/mL)	273
Hemoglobin (g/dL)	8.2
Hematocrit (%)	24
Platelet (10^4^/mL)	23.7
APTT (%)	103
PT-INR	1.01
Fibrinogen (mg/dL)	443
Total protein (g/dL)	6.4
Total bilirubin (mg/dL)	0.4
Aspartate aminotransferase (U/L)	13
Alanine aminotransferase (U/L)	6
Creatinine (mg/dL)	0.42
Sodium (mEq/L)	135
Potassium (mEq/L)	4.1
Chloride (mEq/L)	104
C-reactive protein (mg/dL)	0.4

APTT: activated partial thromboplastin time.

PT-INR: prothrombin time-international normalized ratio.
